# Analysis of the efficacy, safety, and cost of alternative forms of creatine available for purchase on Amazon.com: are label claims supported by science?

**DOI:** 10.1016/j.heliyon.2022.e12113

**Published:** 2022-12-06

**Authors:** Guillermo Escalante, Adam M. Gonzalez, Dean St Mart, Michael Torres, Jacob Echols, Mariesha Islas, Brad J. Schoenfeld

**Affiliations:** a California State University San Bernardino, USA; b Hofstra University, USA; c Supplement Needs, United Kingdom; d CUNY Lehman College, USA

**Keywords:** Supplementation, Creatine monohydrate, Multi-ingredient, Functional ingredients

## Abstract

Creatine monohydrate (CM) is an established and effective dietary supplement, but it is not the only form of creatine. We analyzed forms of creatine for sale on Amazon.com" title = "http://Amazon.com">Amazon.com and evaluated if the advertised claims are supported by the available scientific evidence. We also analyzed the cost per gram of the forms of creatine. A total of 175 creatine supplements were included and we reported the total creatine content per serving, form(s) of creatine in products, product claims, and prevalence of products third party certified. The identified products contained 16 forms of creatine other than CM. The prevalence of products containing functional ingredients with CM or forms of creatine was 29.7%, and the prevalence of products containing blends of different forms of creatine was 21.7%. Only 8% of products were third party certified. The products using only CM (n = 91) had a mean price per gram of $0.12 ± 0.08, whereas products using only other forms of creatine (n = 32) had a mean price per gram of $0.26 ± 0.17. Approximately 88% of alternative creatine products in this study are classified as having limited to no evidence to support bioavailability, efficacy, and safety.

## 1 Introduction

Creatine (N-(aminoiminomethyl)-N-methyl glycine) is a naturally occurring nitrogenous compound derived from the amino acids glycine and arginine. Creatine is synthesized endogenously by the transfer of the amidino group of L-arginine to the N^α^-amine group of L-glycine that is catalyzed by L-arginine-glycine amidinotransferase to yield ornithine and guanidinoacetate (GAA); GAA is then methylated by guanidinoacetate N-methyltransferase with S-adenosyl methionine to form creatine [5, 23, 35, 37, 52]. Although creatine is not a traditional proteinogenic amino acid [35], it is frequently referred to as an amino acid in the literature [1, 5, 13, 26, 36, 66] because it is a non-proteinogenic amino acid similar to ornithine, citrulline, and homoserine [5, 7, 40, 45]. In a general sense, an amino acid can be any organic compound that contains both an amino group and carboxylic group [40].

Creatine has recently been proposed to be a conditionally essential nutrient for humans due to the need to obtain this nutrient from the diet for normal growth, development, and health [47]. Roughly half of the daily creatine requirements for an average person (∼1–1.5 g) are produced in the kidneys, liver, pancreas, and brain [8]. However, the remaining creatine content must be supplied by the diet from foods such as meat, beef, and fish [35, 36, 47] or from dietary creatine supplementation. Creatine supplements are among the most popular ergogenic aids used by athletes as hundreds of studies have consistently shown that supplementation leads to improvements in high intensity exercise performance to enhance strength, muscle mass, power production, and sprint performance [66]. Other research on creatine has also shown the potential benefits of supplementation on injury prevention, improved exercise recovery, enhanced tolerance to exercise in the heat, improved rehabilitation outcomes, brain and spinal cord neuroprotection, ischemic heart disease, aging, and other health conditions/special populations [36, 37]. As a result, it has been reported that Americans consume over four million kilograms of creatine per year with the worldwide use being significantly higher [36]. Indeed, the creatine market is expected to increase from $360 million in 2020 to $520 million by 2024 [75].

The type of creatine primarily used in research to establish its safety and efficacy has traditionally been a micronized creatine monohydrate (CM) made by AlzChem in Germany under the brand name Creapure®; this brand of CM has been reported to produce CM that is 99.9% pure [1, 26, 35, 36]. Thus, it is typically considered the gold standard. CM may also come from sources produced in China that have different starting materials such as sarcosinates and S-alkylisothiourea (as opposed to sodium sarcosinate, acetic acid, and cyanamide) and have been reported to produce more contaminants due to the methods used to manufacture it [1, 35, 53].

CM itself is marketed and sold in various forms such as micronized CM (CM with smaller mesh sized particles), buffered CM (CM “pH-corrected” to a higher pH), effervescent CM, serum CM, and Creabev® (described as a soluble and stable form of CM to enhance stability in fluid); however, some of these have no evidence of being effective and/or superior forms of CM despite the marketing claims made by supplement manufacturers [35]. In addition to other forms of CM marketed and sold in the United States that have not been as thoroughly studied as Creapure®, other novel or alternative forms of creatine that are not CM such as creatine salts [e.g., creatine citrate, creatine maleate, creatine pyruvate, creatine orotate, creatine hydrochloride (HCL), etc.], creatine derivatives [e.g., creatine ethyl ester (CEE)], creatine precursors [e.g., guanidinoacetic acid (GAA), creatinol-o-phosphate (COP)], and creatine dipeptides (e.g. creatyl-l-leucine) are commonly sold as creatine-containing supplements either as individual products or as part of multi-ingredient products [1, 9, 26, 35, 36, 58]. Similar to some types of CM, research on the efficacy and safety of these novel/alternative forms of creatine is also lacking as relatively few studies have been performed to study their safety and/or effectiveness [26, 35]. To this point, a recent review by Fazio et al. compared the effects of magnesium-creatine chelate, creatine citrate, creatine malate, CEE, creatine nitrate, and creatine pyruvate and reported no consistent findings for performance enhancement among the alternative forms of creatine when compared to a placebo [13]. Additionally, Kreider and colleagues published 2 papers in the last 11 years on the bioavailability, efficacy, safety, and regulatory status of novel forms of creatine and pointed to the lack of scientific evidence to support the manufacturers’ marketing claims [26, 35]. Several other publications such as those by the International Society of Sports Nutrition (ISSN) over the last 15 years [1, 9, 36] also highlight the lack of scientific support for novel/alternative forms of creatine. Nevertheless, it appears to be common practice for creatine supplement manufacturers to use evidence from studies conducted on effective sources of CM such as Creapure® to substantiate the effects of their particular type of creatine and/or claim that their product is superior to effective sources of CM.

Since online supplement sales have increased over the last several years and are forecasted to make up 25% of all supplement sales by 2024 [42], this investigation analyzed the forms of creatine available for purchase online. Specifically, creatine supplements sold on Amazon.com were investigated because Amazon.com has been reported to make up approximately 77% of all online supplement sales [72]. Thus, the purpose of this investigation was to analyze the types of creatine available for sale on Amazon.com" title = "http://Amazon.com">Amazon.com and to evaluate if the advertised claims are supported by the available scientific evidence. We also sought to analyze the prevalence of products that contain alternative forms of creatine and the cost per gram of different forms of creatine (as compared to CM). Lastly, we identified the creatine products that advertised they are certified for purity/no banned substances by any of the recognized third-party certifying bodies inclusive of Informed Choice, Informed Sport, National Sanitation Foundation (NSF) International, or the Banned Substances Control Group (BSCG).

## 2 Methods

A search for the product “creatine” was performed on Amazon.com on 1/28/2022 that yielded a total of seven web pages of products for analysis. As the information used for analysis is publicly available and required no involvement of human or animal subjects, approval from an Institutional Review Board was not necessary. Multi-ingredient pre-workout or post-workout supplements, as well as products not specifically marketed as a creatine supplement, were excluded from the analysis; however, products marketed as creatine with other added functional ingredients (e.g., glutamine, citrulline, taurine, etc.) were included. A total of 204 supplements that were marketed as creatine were identified in the search. After identifying duplicate products or products that were the same but sold in various sizes, a total of 175 supplements were included in this study. In cases where several sizes of the same product were identified, the larger size was used for analysis as it was the most cost effective per serving size.

The product label and Amazon.com sales page were analyzed for each supplement to obtain the following information: the total creatine content per serving, form(s) of creatine in formula, product description/claims, and if the product was advertised as third party tested by Informed Choice, Informed Sport, NSF International, or BSCG. In some cases, creatine was provided in an undisclosed quantity as part of a proprietary blend, and therefore, specific amounts were not available. When applicable, the price per gram of creatine was calculated and included in the analysis. If a form of creatine was included in a product without any other functional ingredients, it was deemed a stand-alone creatine ingredient. Added functional ingredients included other dietary ingredients purported to exert ergogenic properties listed in the nutrition facts section of the label. Ingredients listed as “other ingredients” such as preservatives, sweeteners, electrolytes, artificial flavoring agents, and food coloring were not included in the analysis.

In order to evaluate if the advertised claims are supported by the available scientific evidence, the recent review article by Kreider et al. was utilized because it classifies various forms of creatine based on the evidence to support bioavailability, efficacy, and safety as: (a) Strong evidence, (b) Some evidence, or (c) No evidence [35]. To the authors’ knowledge, this is the only article that specifically classifies alternative forms of creatine categorically similar to other dietary supplements discussed in the comprehensive 2018 International Society of Sports Nutrition exercise and sport nutrition research and recommendations manuscript [32]. While the Kreider et al. [35] review article has not been validated and the recommendations therein are not specifically adopted or enforced by the Food and Drug Administration (FDA) or the Federal Trade Commission (FTC) that oversee dietary supplements and advertising of dietary supplements, it does provide a detailed summary of the research (or lack thereof) on alternative forms of creatine. We recognize this is an inherent limitation of this study and we disclose some limitations of this reference in our discussion. Regardless, the classification system serves as a logical categorization of the evidence available of alternative forms of creatine.

### 2.1 Statistical analysis

Basic descriptive and frequency analysis was performed for the forms of creatine included in the supplements analyzed in this study. The overall prevalence, prevalence in undisclosed/listed quantities, and prevalence as a stand-alone creatine ingredient were determined for each form of creatine. Prevalence of third-party certification was also reported. The listed quantity (mean ± standard deviation) along with price per gram of creatine were reported for each form of creatine when included as a stand-alone creatine ingredient. The prevalence of each form of creatine being included as part of a blend of different forms of creatine was also reported. Product descriptions were analyzed for common marketing claims associated with each form of creatine. Lastly, alternative forms of creatine were classified based on evidence to support bioavailability, efficacy, and safety as: (a) Strong evidence, (b) Some evidence, or (c) No evidence.

## 3 Results

One hundred and seventy-five creatine products were included in this analysis. Products contained 16 different forms of creatine that were not CM (i.e., Creapure®, other micronized CM, or other forms of CM excluding buffered creatine that was categorized separately). The prevalence of products containing other functional ingredients (e.g., betaine anhydrous, beta-hydroxy-beta-methylbutyrate, carbohydrates, taurine, citrulline, glutamine, etc.) with CM or alternative forms of creatine was 29.7%. The prevalence of products containing blends of different forms of creatine was 21.7%, with blends ranging from 2 to 11 different types of creatine. The prevalence of products that were third party certified was 8%. The products with no other functional ingredients using only CM (n = 91) had a mean price per gram of creatine of $0.12 ± 0.08, whereas products with no other functional ingredients using alternative forms of creatine (n = 32) had a mean price per gram of creatine of $0.26 ± 0.17. A summary of overall market and market prices are described in Table 1.

**Table 1:** Overall market and market prices of creatine products available on Amazon.

Overall Market	
Creatine products examined by this study (n)	175
Prevalence of products containing other functional ingredients with creatine monohydrate or forms of creatine (%)	29.7
Prevalence of blends of different forms of creatine (%)	21.7
Total creatine monohydrate or form of creatine content per serving (mg)	4239 ± 1420
**Market Prices**	
	**Price per gram of creatine (Mean ± SD)**
All products (n = 175)	$ 0.19 ± 0.14
Products with other functional ingredients (n = 52)	$ 0.27 ± 0.14
Products with no other functional ingredients (n = 123)	$ 0.15 ± 0.12
Products with no other functional ingredients using only creatine monohydrate (n = 91)	$ 0.12 ± 0.08
Products with no other functional ingredients using other forms of creatine (n = 32)	$ 0.26 ± 0.17

**Figure 1:**
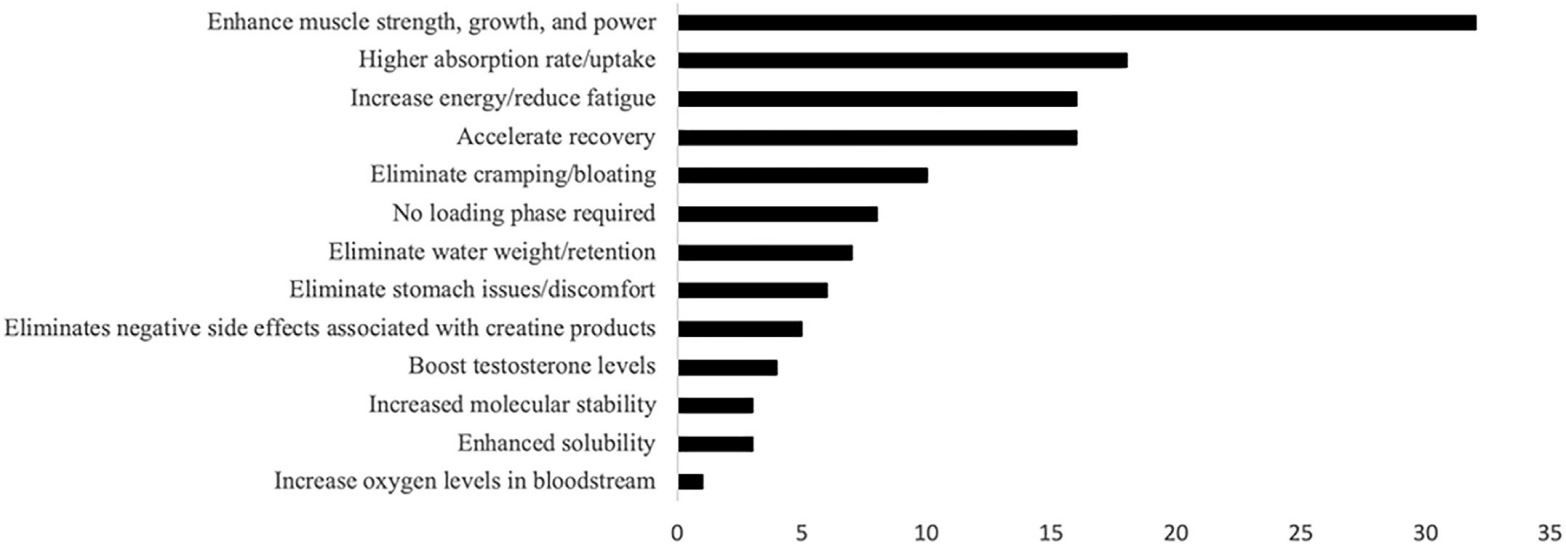
Common marketing claims for alternative forms of creatine.

The prevalence and quantities of different forms of creatine included in the Amazon.com search for all creatine products (n = 175) and creatine products that do not contain other functional ingredients (n = 123) are provided in Table 2 and Table 3, respectively. Additionally, Table 3 includes the price per gram of creatine for each type of creatine when sold as a stand-alone ingredient. Table 4 includes similar prevalences and quantities of different forms of CM (i.e., Creapure®, other micronized CM, microencapsulated, and other forms of CM not specified as Creapure® excluding buffered creatine that was categorized separately) found in the Amazon.com search for creatine products that do not contain other functional ingredients and contain only a type of CM (n = 91). The most common marketing claims for alternative forms of creatine that are not CM are depicted in Figure 1. Lastly, Table 5 classifies the various forms of creatine available for purchase on Amazon.com" title = "http://Amazon.com">Amazon.com as either strong evidence, some evidence, or no evidence to support bioavailability, efficacy, and safety for each type of creatine as categorized by Kreider et al. [35] (if the information was provided in that article); it also provides a brief summary of available studies that have been conducted on each ingredient.

**Table 2:** Prevalence and quantities of different forms of creatine included in creatine products (n = 175).

Form of Creatine	Overall Prevalence (%)	Prevalence in Undisclosed Quantity (%)	Prevalence in Listed Quantity (%)	Prevalence as Stand-alone Creatine Ingredient (%)	Listed quantity (Mean ± SD) as a Stand-alone Creatine Ingredient (mg)	Prevalence as Part of a Blend of Different Forms of Creatine (%)
Creatine Monohydrate∗	82.3	9.7	72.6	65.1	4503 ± 1067	16.5
Buffered or KreAlkalyn Creatine Monohydrate	6.9	1.7	5.1	5.1	1839 ± 718	1.7
Di-Creatine Malate	6.9	5.7	1.1	0	N/A	6.8
Creatine Gluconate	2.9	1.7	1.1	0.6	3000	2.2
Creatine Anhydrous	4	3.4	0.6	0.6	3000	3.4
Magnesium Creatine Chelate	13.7	8	5.7	1.7	3950 ± 2051	12.0
Creatine Phosphate	1.7	1.7	0	0	N/A	1.7
Creatine Alpha-ketoglutarate	7.4	4.6	2.9	0	N/A	7.4
Creatine Pyruvate	8.6	2.9	5.7	0	N/A	8.5
Creatine Citrate	4.6	4	0.6	0	N/A	4.5
Creatine Hydrochloride	10.9	2.3	8.6	3.4	1350 ± 627	7.4
Free Acid Creatine	0.6	0	0.6	0	N/A	0.5
Tri-Creatine Malate	2.9	2.3	0.6	0	N/A	2.8
Creatine Ethyl Ester	2.2	2.3	0.6	0	N/A	2.8
Creatine Nitrate	0.6	0	0.6	0	N/A	0.5
Creatine Ethyl Ester Malate	1.1	1.1	0	0	N/A	1.1
Creatinol-O-Phosphate	1.7	1.7	0	1.1	N/A	0.5

∗ Creapure®, micronized, and other types of creatine monohydrate.

**Table 3:** Prevalence and quantities of different forms of creatine included in creatine products that do not contain other functional ingredients (n = 123).

Form of Creatine	Overall Prevalence (%)	Prevalence in Undisclosed Quantity (%)	Prevalence in Listed Quantity (%)	Prevalence as stand-alone creatine ingredient (%)	Listed quantity (Mean ± SD) as a stand-alone creatine ingredient (mg)	Prevalence as part of a blend of different forms of creatine (%)	Price per gram of creatine when sold as stand-alone ingredient ($)
Creatine Monohydrate∗	85.4	4.9	80.5	74.0	4469 ± 1110	11.3	$ 0.12 ± 0.08 (n = 91)
Buffered or KreAlkalyn Creatine Monohydrate	7.3	0.8	6.5	6.5	1819 ± 764	0.8	$ 0.35 ± 0.20 (n = 8)
Di-Creatine Malate	2.4	2.4	0	0	N/A	2.4	N/A
Creatine Gluconate	0.8	0	0.8	0.8	3000	0	$ 0.17 (n = 1)
Creatine Anhydrous	1.6	0.8	0.8	0.8	3000	0.8	$ 0.22 (n = 1)
Magnesium creatine chelate	6.5	3.3	3.3	0.8	2500	5.6	$ 0.32 (n = 1)
Creatine Phosphate	0	0	0	0	N/A	0	N/A
Creatine alpha-ketoglutarate	5.7	1.6	8.1	0	N/A	5.6	N/A
Creatine Pyruvate	9.8	1.6	8.1	0	N/A	9.7	N/A
Creatine Citrate	2.4	1.6	0.8	0	N/A	2.4	N/A
Creatine Hydrochloride	8.9	0.8	8.1	2.4	917 ± 289	6.5	$ 0.55 ± 0.08 (n = 3)
Free acid creatine	0.8	0	0.8	0	N/A	0.8	N/A
Tri-Creatine Malate	1.6	0.8	0.8	0	N/A	1.6	N/A
Creatine Ethyl Ester	0.8	0.8	0	0	N/A	0.8	N/A
Creatine Nitrate	0	0	0	0	N/A	0	N/A
Creatine Ethyl Ester Malate	0	0	0	0	N/A	0	N/A
Creatinol-O-Phosphate	0.8	0.8	0	0.8	N/A	0	N/A

∗ Creapure®, micronized, and other types of creatine monohydrate.

**Table 4:** Prevalence and quantities of different forms of creatine monohydrate in creatine products that do not contain other functional ingredients and contain only creatine monohydrate (n = 91).

Type of Creatine Monohydrate (CM)	Overall Prevalence (%)	Prevalence in Undisclosed Quantity (%)	Prevalence in Listed Quantity (%)	Prevalence as stand-alone creatine ingredient (%)	Listed quantity (Mean ± SD) as a stand-alone creatine ingredient (mg)	Prevalence as part of a blend of different forms of creatine (%)	Listed quantity (Mean ± SD) as part of a blend of different forms of creatine (mg)	Price per gram when sold as stand-alone creatine ingredient ($)
Creapure® CM	21	0	21	21	4758 ± 818	0	N/A	$ 0.13 ± 0.11
Micronized CM	34	0	34	34	4676 ± 864	0	N/A	$ 0.09 ± 0.05
Other CM	44	0	44	44	4220 ± 1313	0	N/A	$ 0.13 ± 0.07
Microencapsulated CM	1	0	1	1	5000	0	N/A	$ 0.07

**Table 5:** Classification of evidence to support bioavailability, efficacy, and safety for alternative forms of creatine available on amazon.

Type of Creatine	Level of Evidence	Summary of Evidence Available
Creatine Phosphate	No Evidence	Not Available
Creatine Alpha-Ketogluterate	No Evidence	Not Available
Tri-Creatine Malate	No Evidence	Not Available
Creatine Ethyl Ester Malate	No Evidence	Not Available
Creatine Gluconate	No Evidence	Not Available
Di-creatine Malate	No Evidence	Not Available
Creatinol-O-Phosphate (COP)	No Evidence	Intramuscular and intravenous administration of COP ↑ handgrip performance, but no studies have evaluated if it has any effect on muscle creatine levels or exercise performance [35, 43]
Buffered or KreAlkalyn® Creatine	Some Evidence	Recommended doses and loading/maintenance equivalent doses did not provide greater changes in muscle creatine, body composition, strength, or anaerobic capacity compared to CM [27, 35]
Creatine Pyruvate	Some Evidence	↑ intermittent handgrip exercise of maximal intensity and may have benefits with endurance exercise, but conflicting evidence exists on benefits with endurance exercise [25, 35, 64]
Creatine Citrate	Some Evidence	Studies provide evidence it can increase blood creatine levels in a similar manner as CM and there is some data supporting an ergogenic benefit, but the impact of supplementation has not been assessed on muscle or brain creatine content. No studies currently indicate it is more effective or safe than CM [16, 19, 24, 25, 35, 61]
Creatine Hydrochloride	Some Evidence	This creatine salt should disassociate into creatine and HCL and is bioavailable, but there is no evidence that it is absorbed more effectively than CM in humans, promotes greater muscle creatine retention than CM at same dosages, or that lower doses are more effective than standard CM doses [15, 35, 63, 71]
Creatine Ethyl Ester (CEE)	Some Evidence	Likely that some of the ingredient is degraded into creatinine during normal digestion, but some creatine will be delivered to blood. Supplementation has shown increases in muscle creatine content vs. placebo after 27 days of supplementation, but less than those taking CM. Supplementation during resistance training also showed increases in body weight and leg press strength while body fat decreased, but results were not better than using CM. No evidence that ingesting recommended dosages of supplement is more effective than recommended dosages of CM [2, 35, 59]
Creatine Nitrate	Some Evidence	Some evidence to support it is a bioavailable source of creatine in proportion to the amount of creatine delivered during the loading phase, but not more bioavailable than CM when equivalent doses are ingested. Some evidence to suggest supplementation provides an ergogenic effect for muscle endurance, but since it was unrelated to changes in muscle creatine content the benefit may come from the nitrate as opposed to creatine. Remains to be determined if supplementation has any additional benefit than simply co-ingesting CM with a source of nitrate [12, 17, 28, 29, 35, 50]
Magnesium Creatine Chelate	Some Evidence	No evidence it is more bioavailable, efficacious, and/or safer than CM and limited evidence it is as effective as CM to increase strength, power, and muscle endurance [6, 35, 55, 68]
Creatine Anhydrous	Not Provided	No data available, but this compound is 100% creatine and likely has similar effects to CM
Free Acid Creatine	Not Provided	Not available

## 4 Discussion

A total of 175 products marketed as creatine were available for sale by searching “creatine” on Amazon.com. Of these, 16 forms of creatine that were not Creapure®, other micronized CM, or other forms of CM (excluding buffered creatine that was categorized separately) were available for sale as either stand-alone products, creatine blends, and/or creatine (monohydrate or other forms of creatine) mixed with other functional ingredients. Interestingly, the products containing only CM (n = 91) had a mean price per gram of $0.12 ± 0.08, whereas the mean price per gram of creatine for other stand-alone forms of creatine (n = 32) was ∼116% higher at $0.26 ± 0.17. This could be due to the higher costs associated with obtaining alternative forms of creatine and/or for companies to increase profit margins.

Only 8% of the creatine products in this study advertised they are third party certified for purity and/or banned substances by any of the recognized third-party bodies inclusive of Informed Choice, Informed Sport, NSF International, or BSCG. While this is strictly a voluntary practice performed by some supplement manufacturers to be transparent about the purity and safety of their ingredients/products, it is not possible to rule out that some creatine supplements in this study may have impurities and/or banned substances (e.g., steroid precursors) in their products that may promote performance enhancement benefits above and beyond what a creatine containing product may yield. Indeed, concerns with adverse events and failed doping tests due to contaminated dietary supplements has been a topic of discussion in the peer-reviewed literature [41], and a recent article reported that 875 of 3132 supplements analyzed (∼28%) contained undeclared substances inclusive of anabolic-androgenic steroids [33].

In addition to the fact that alternative forms of creatine cost ∼116% more per gram of creatine than CM, our analysis revealed that a common practice among creatine supplement manufacturers is to use evidence from studies conducted on effective sources of CM such as Creapure® to substantiate the effects of their particular type of creatine and/or claim that their product is superior to other sources of CM. While all of the alternative forms of creatine found available for purchase on Amazon.com market their products this way, there is minimal scientific evidence to substantiate these claims. This is in direct contrast with the recommendation recently made by Kreider et al. when the authors state that claims about a form of creatine should be based on the research conducted on that specific type of creatine at the appropriate doses and not based on assumptions or marketing hyperbole [35].

A total of 43.75% (n = 7) of creatine related compounds found in this study have been classified as having no evidence to support bioavailability, efficacy, and safety, 43.75% (n = 7) have limited evidence, and 12.5% (n = 2) were not classified in the paper by Kreider et al [35]. Of the 2 types of creatine that were not classified by Kreider et al. [35], creatine anhydrous is actually 100% creatine, but due to the high temperature required to dry the compound it is reported to contain larger quantities of creatinine [26, 35]. However, while this has not been tested to the authors' knowledge, its effects are likely similar to those of CM. The other compound not classified, free acid creatine, has not been tested to the authors’ knowledge and thus would merit a classification of no evidence; however, it is unknown if this type of creatine may be creatine HCL but simply called free acid creatine by the manufacturer.

It should be noted that although some of the alternative forms of creatine are classified as having some evidence to support their bioavailability, efficacy, and safety, none have been to be superior to CM in terms of increasing muscle creatine content [35] despite the commonly used marketing claims/marketing hyperbole shown in Figure 1. The only caveats to some alternative forms of creatine being superior to CM are that CM has moderately poor water solubility, is relatively unstable in aqueous solutions because of its tendency to cyclize into creatinine, and is dependent on the finite capacity of creatine transporters for absorption [35, 65]. As a result, the development of alternative forms of creatine to overcome these limitations and/or different ways to consume creatine such as mixing with lower pH beverages continue to be explored [35, 65]. Hence, despite a lack of supporting evidence that the alternative forms of creatine found in this study are more effective in increasing tissue creatine content as compared to CM, some forms such as creatine salts (e.g., creatine HCL) are superior to CM in terms of solubility when mixed in a beverage [15].

Since the CM manufactured in Germany by AlzChem (Creapure®) has been the primary source of creatine used in hundreds of clinical trials to test its safety and effectiveness [35], the scientific community is generally aware that it is considered the gold standard of creatine. However, Creapure® is not the only type of creatine that is marketed and sold on Amazon.com and is widely available from other online and brick-and-mortar retailers. As such, although the words “creatine supplementation” are typically understood to mean CM supplementation in the scientific literature [35], it does not specifically mean Creapure® supplementation (or CM in general) that has been clinically tested since other forms of CM (e.g., other CM powders, CM lozenges, buffered CM, creatine serum, Creabev®, etc.) have been used in research and not all have been reported to be as effective as Creapure®. Indeed, some forms of CM (e.g., microencapsulated CM) have not been tested at all, and others such as creatine serum have been reported to be ineffective altogether [20, 35, 38]. Furthermore, the term “creatine supplementation” is clearly not considered exclusive to mean CM for companies that sell any form of creatine nor for the general public without a formal scientific background. As such, when using the term “creatine supplementation” outside of the scientific community, it is important to take into account that this may include one of the many forms of CM available for purchase or forms of creatine that have only been reported in patents or the scientific literature. As an example, even the United States Department of Defense Dietary Supplement Resource on Creatine Supplement Basics recognizes CEE, creatine magnesium chelate, creatine HCL, and creatine malate (among others) as forms of creatine [74].

Kreider et al. attempted to provide recommendations regarding the type of research required to validate that a form of creatine is a bioavailable, effective, and safe source of creatine in review papers from 2011 and 2022 [26,35]; however these recommendations have not been adopted by supplement manufacturers, nor have they been adopted or enforced by the Food and Drug Administration (FDA) or the Federal Trade Commission (FTC) that oversee dietary supplements and advertising of dietary supplements. If this was the case, supplement manufacturers and retailers such as Amazon.com would likely have more stringent policies and the marketing practices of alternative forms of creatine would be altered and/or disallowed. Indeed, Kreider et al. discuss that the scientific basis and regulatory status of alternative forms of creatine is not clear and continues to be common in the sports supplement industry [35]. This is in spite of the 2011 review paper on novel forms of creatine co-authored by Kreider and colleagues [26], other publications that have discussed alternative forms of creatine over the last 15 + years [1, 9, 13, 36, 58, 65], and the fact that some forms of creatine are still available today as stand-alone products (e.g., creatine serum) that have been reported to be ineffective in research studies [3, 20, 38].

Some of this may in part be a result of the lack of requirements for supplement manufacturers to provide scientific evidence that their products are effective before coming to market [11]. Although government agencies encourage companies to conduct scientific investigations to evaluate the efficacy, safety, and value of dietary supplements [76], there is no legal requirement or enforcement for them to do so [11]. As such, companies frequently use studies performed on ingredients in the same general space as theirs (e.g., creatine supplement companies may use research performed on the clinically tested Creapure®) to provide support that their products are effective. In situations where supplement companies claim their product to be superior to others, it is likely marketing hyperbole that is commonly used as a marketing strategy by many companies worldwide due to its effectiveness in improving advertising outcomes [60].

While the use of marketing hyperbole within the dietary supplement industry has been controversial for decades [21], products that contain dietary supplements (including forms of creatine) continue to use marketing hyperbole despite some of the marketing/advertising guidelines set forth by the FTC [69]. This is not always without consequence since the FDA and/or FTC have filed cases challenging health claims and safety from questionable marketing practices of manufacturers that make/sell dietary supplements [44, 70], but it is nonetheless prevalent in the industry [22]. Many other dietary supplements that are classified as having little to no evidence to support efficacy and/or safety per the International Society of Sports Nutrition (ISSN) exercise and sports nutrition review article (e.g., arginine, carnitine, glutamine, ribose, medium chain triglycerides, human growth hormone secretagogues, etc.) [31] are available for purchase on internet retail sites despite their lack of support for being effective and/or safe. Furthermore, dietary supplements that were once thought to be effective but now lack scientific support when adequate dietary protein is consumed [e.g., branched chain amino acids (BCAAs)] are also still available for purchase in the marketplace, including Amazon.com" title = "http://Amazon.com">Amazon.com, as stand-alone products or as part of multi-ingredient products. Indeed, marketing hyperbole appears to be the rule rather than the exception since the burden of proof typically required to prove deceptive advertising can be difficult to provide. For example, if there is a lack of consensus in the scientific literature about the research supporting the effectiveness of a particular product because of conflicting studies, then the plaintiff sometimes cannot meet its burden of proof that a company is deceiving customers [39]. In other words, the plaintiff's criticism of the significance of the studies presented by the defense do not necessarily prove that a manufacturer's claims are false. As a result, there is a lack of consistent consequences that supplement companies may face from using marketing hyperbole as recently occurred in a false advertising case of a creatine-glutamine supplement: the court ruled in favor of the supplement company stating that the company was in the right with the structure and function claims related to the benefits of glutamine despite the low dosage of glutamine found in the product [73].

It is also important to comprehend that it often takes several years and many studies on a product/ingredient to determine if it is effective and/or safe. Even CM, which now has hundreds of studies to substantiate its effectiveness, was not always shown to be effective when it first came to market. For example, some initial studies performed on animals with CM did not successfully increase muscle creatine content [56]; however, this is due to species-specific differences in creatine metabolism/storage and results of the data in animals not always carrying over to humans [34, 35]. Even in human studies, the data on the effects of CM supplementation is often variable between individuals, populations, and studies. Indeed, a relatively substantial portion of the population (∼30%) are deemed “non-responders” to creatine supplementation, which may influence mean results of a given sample [62]. In one relatively early review paper on the ergogenic benefits of CM supplementation in 2003, eleven studies were reported where CM had no ergogenic effects [4]. Similarly, CM supplementation has been shown to augment brain creatine content in some studies, but not others [14].

Since few studies on most alternative forms of creatine have been published in the peer reviewed literature, the evidence available is preliminary at best. This is one major limitation of the Kreider et al. [35] reference we used to classify alternative forms of creatine in this investigation since that review paper does not take this factor into consideration. As an example, research on the creatine precursor guanidinoacetic acid (GAA) is perhaps the most comprehensive with many animal and human studies completed thus far; however, studies on this alternative form of creatine began over 70 years ago [14, 46, 49]. Preliminary evidence suggests that GAA may be as effective, if not more effective, than CM supplementation as summarized in a review paper by Ostojic et al. [49]. Thus, just because a few preliminary studies, especially those conducted in animals or in human studies with methodological limitations and/or potential conflicts of interest, show that an alternative form of creatine is not effective does not mean it will be demonstrated to be ineffective in the future. This is especially apparent in the evolving field of sports nutrition where products/ingredients that were once believed to be less effective than others have been shown otherwise; conversely, some products/ingredients that were once believed to be potentially effective have been shown to have questionable benefits as research evolved. For example, plant-based protein supplements were once believed to be inferior to animal-based protein supplements, but newer research has challenged this notion if they are manufactured to contain similar quantities of leucine and other essential amino acids [30]. On the other hand, supplementation with BCAAs was once accepted as potentially beneficial, but recent research indicates they offer no ergogenic benefits provided sufficient total daily protein intake [54, 67]. In regards to the lack of ergogenic benefits to supplementing with BCAAs for all individuals, supplement companies still sell them as either stand-alone products or as part of a multi-ingredient product on Amazon.com and other retail stores. As such, it is challenging to monitor and enforce policies since the science of sports nutrition rapidly evolves. Alternatively, this could be viewed as marketing hyperbole that seems to be common practice in the dietary supplement industry.

One of the recommendations put forth by Kreider et al. to describe the data and research needed to substantiate claims that an alternative form of creatine is a bioavailable, effective, and safe source of creatine for dietary supplements states that the first step in accomplishing this is to determine whether the form of creatine contains a creatine molecule [35]. While this may seem like a logical first step, it can be extremely restrictive as some alternative forms of creatine do not contain a complete creatine molecule that have been shown to have positive supplementation effects. Indeed, this restrictive definition serves as another limitation of our reference for classification of alternative forms of creatine. As an example, the creatine precursor GAA does not contain a complete creatine molecule but has been shown to have similar (or perhaps superior) effects compared to using CM [49]. Similarly, the creatine derivative creatine ethyl ester (CEE) does not have a complete creatine molecule since it is a chemically altered form of creatine [59]. Regardless, CEE is recognized as a form of creatine by the United States Department of Defense Dietary Supplement Resource and supplementation with CEE has been shown to increase total muscle creatine content, body weight, and leg press strength while simultaneously decreasing body fat [2, 59, 74]. Although CEE has not been reported to be as effective as CM supplementation, some research indicates that it is capable of some favorable effects [2, 59]. Lastly, creatinyl amino acids do not contain a complete creatine molecule, but a preliminary study by Burov et al. suggests they may possess neuroprotective properties similar to creatine but with a potentially enhanced penetration of the cell membrane and blood brain barrier; this could prove to be important for the treatment of acute and chronic neurological diseases such as stroke, traumatic brain injury, and hereditary creatine transporter deficiency [10]. The study by Burov et al. also suggests that creatinyl amino acids can reach the target cells without significant decomposition and undergo enzymatic cleavage near the sites of action [10]. Indeed, a follow-up study on creatinyl amino acids by Garbati et al. demonstrated that two of these alternative forms of creatine [creatine-glycine ethyl ester (CGE) and creatine-ethyl amide (CNE)] were able to significantly increase brain creatine content better than CM when the creatine transporter was blocked and slightly less than CM when the creatine transporter was not blocked [18]. While conflicting evidence on the bioavailability of a similar alternative form of creatine [creatyl-l-leucine (CLL)] has been reported by da Silva, the author did acknowledge that it could not be concluded CLL was not absorbed due to the limit of detection of the high-performance liquid chromatography assay [57]. Thus, this calls into question the strength of the conclusive statement by Kreider et al. that creatine amide bonds likely do not increase creatine levels in the blood or tissues [35]. Although beyond the scope of this paper, significantly more research is required, ideally in humans (as opposed to animals), to study the pharmacokinetics, pharmacodynamics, and efficacy of alternative forms of creatine such as CEE, CLL, and other creatinyl amino acids.

Kreider et al. also state that a type of creatine that does not increase muscle and/or brain creatine content in humans should not be considered as a viable source of creatine [35]. While this is an idealistic recommendation that perhaps should be adopted, careful evaluation/assessment of the pharmacokinetics and pharmacodynamics of these forms of creatine are required because alternative forms of creatine likely have different pharmacokinetics and pharmacodynamics than CM but still have the capacity to yield favorable creatine-like benefits. Indeed, the main purpose of many of these alternative forms of creatine is to provide a product that overcomes the few limitations that CM has, as previously stated. For example, the creatine precursor GAA was reported to have a 3.3-fold higher increase in tissue creatine levels as compared with using CM and the researchers hypothesized this may be due to the preferable uptake of GAA by target tissues via various mechanisms theoretically available for GAA transport, as compared with somewhat limited transport capacity of creatine, and/or complete/near complete tissue methylation of GAA to creatine [48]. The authors also stated that while creatine is mainly transported via specific creatine transporters, dietary GAA could be imported through additional delivery channels in tissue such as the brain [48]. GAA does not enter the body as creatine; instead, it enters the body as GAA before it is absorbed in the gut and then methylated to become creatine. Since GAA must first convert to creatine in the body, plasma concentrations of creatine do not peak until ∼2 h following ingestion of GAA; on the other hand, peak plasma concentrations of creatine peak earlier after ∼1 h following ingestion of CM [51].

Although GAA and CM both heavily rely on creatine transporters to go to target tissues such as the brain and muscle, other alternative forms of creatine such as creatinyl amino acids are primarily designed to eliminate the need for creatine transporters to cross biological membranes of tissues, especially the brain [18]. This may be accomplished by the creatinyl amides serving as their own creatine carrier or the compounds being more lipophilic than creatine itself [18]. As stated previously, other potential mechanisms of action need to be explored, but that is beyond the scope of this paper. However, it is clear that more research is required on the pharmacokinetics and pharmacodynamics of alternative forms of creatine before they are considered ineffective.

The current reality of the practices within dietary supplement companies that manufacture any form of creatine is that they are not in line with the recommendations set forth by Kreider et al. in their review papers [26, 35]. The current dietary supplement regulations enforced by the FDA and the FTC are relatively ambiguous and relaxed as compared to the recommendations set forth by Kreider et al. [26, 35]. Indeed, while the FDA oversees dietary supplements, they are regulated as food and not as drugs [11]. According to the FDA, “unlike drug products that must be proven safe and effective for their intended use before marketing, there are no provisions in the law for FDA to ‘approve’ dietary supplements for safety or effectiveness before they reach the consumer. Under the Dietary Supplement Health and Education Act (DSHEA), once the product is marketed, the FDA has the responsibility for showing that a dietary supplement is ‘unsafe,’ before it can take action to restrict the product's use or removal from the marketplace” [11]. Although alternative forms of creatine that entered the marketplace after passing of the DSHEA on 15 October 1994 are considered new dietary ingredients (NDI) and manufacturers are expected to notify the FDA, the FDA only indicates if the NDI is considered relatively safe for human consumption and does not confirm the efficacy of the NDI [35]. Thus, safety is a larger concern for the FDA with dietary supplements as opposed to efficacy and/or bioavailability. As such, revisions to what is required from dietary supplement companies and regulating/enforcing these requirements would be needed before the recommendations set forth by Kreider et al. [26, 35] become a reality. Until such revisions occur and/or more guidance is provided by governing bodies, some level of marketing hyperbole will likely remain common practice among companies that sell any form of creatine and other dietary supplements.

Our study is not without limitations as we only investigated products sold on Amazon.com. As such, our sample is not representative of all supplements sold online directly to consumers nor of those sold in brick and mortar stores. We also only analyzed the information as reported on the labels of the creatine products; this may or may not be accurate. Future research in this area could physically analyze the purity, safety, and effectiveness of alternative forms of creatine products as sold to consumers. Although this would require significant external funding to purchase/study/analyze the hundreds of creatine products that exist, it would be helpful to gain this knowledge. Lastly, our manuscript relies on categorizing the evidence available on alternative sources of creatine on a recent critical review paper that has not been specifically validated. Some of the limitations of this reference are further highlighted in previous sections of this discussion.

## 5 Conclusion

In conclusion, all alternative forms of creatine found in this investigation utilize marketing hyperbole to sell their products. This practice appears to be common among the dietary supplement industry in general [22]. The alternative forms of creatine in this study cost ∼116% more than other effective forms of CM. Despite the higher prices, ∼88% of alternative creatine products found in this study have been classified as having limited to no evidence to support bioavailability, efficacy, and safety. Although research on the effectiveness of most alternative forms of creatine is currently limited, more research is warranted before conclusive statements can be made about their lack of effectiveness as creatine precursors such as GAA, and other alternative forms of creatine, have shown some promise. While none of the forms of creatine in this investigation have current support to show that they are more effective increasing tissue creatine content as compared to CM, some forms of creatine such as creatine salts (e.g., creatine HCL) are superior to CM in terms of solubility when mixed in a beverage.

## Declarations

### Author contribution statement

Guillermo Escalante: Conceived and designed the experiments; Performed the experiments; Analyzed and interpreted the data; Contributed reagents, materials, analysis tools or data; Wrote the paper.

Adam Gonzalez: Conceived and designed the experiments; Analyzed and interpreted the data; Contributed reagents, materials, analysis tools or data; Wrote the paper.

Dean St Mart: Conceived and designed the experiments; Wrote the paper.

Michael Torres; Jacob Echols: Conceived and designed the experiments; Performed the experiments; Wrote the paper.

Mariesha Islas: Performed the experiments; Wrote the paper.

Brad J. Schoenfeld: Conceived and designed the experiments; Analyzed and interpreted the data; Wrote the paper.

### Funding statement

This research did not receive any specific grant from funding agencies in the public, commercial, or not-for-profit sectors.

### Data availability statement

Data will be made available on request.

### Declaration of interest's statement

The authors declare the following conflict of interests.

GE serves as a scientific consultant to Bang and serves/has served as an expert witness on current and past cases related to creatine. GE also serves on the board of directors for Bang. AMG declares that he serves as a scientific

advisor for Shifted LLC, a manufacturer of sports supplements. BJS formerly served on the scientific advisory board of Dymatize Nutrition, a manufacturer of sports supplements. DSM is an employee of the UK supplement brand.

Supplement Needs and serves as the product formulator for the company; he does not have any formulas or intellectual property with any novel forms of creatine and the company only stocks a single ingredient source of creatine

monohydrate. The other authors report they have no conflicts of interest.

### Additional information

No additional information is available for this paper.
